# Calcitonin gene-related peptide is a key neurotransmitter in the neuro-immune axis

**DOI:** 10.3389/fnins.2014.00023

**Published:** 2014-02-14

**Authors:** Bakri M. Assas, Joanne I. Pennock, Jaleel A. Miyan

**Affiliations:** ^1^Translational Medicine, Faculty of Medical and Human Sciences, The University of ManchesterManchester, UK; ^2^Department of Immunology, Faculty of Applied Sciences, King Abdulaziz UniversityJeddah, Saudi Arabia; ^3^Neurosciences, Faculty of Life Sciences, The University of ManchesterManchester, UK

**Keywords:** CGRP, TRPV1, neuroimmunology, TNF-alpha, C fibers, gut-brain axis

## Abstract

The question of how the neural and immune systems interact in host defense is important, integrating a system that senses the whole body with one that protects. Understanding the mechanisms and routes of control could produce novel and powerful ways of promoting and enhancing normal functions as well as preventing or treating abnormal functions. Fragmentation of biological research into specialities has resulted in some failures in recognizing and understanding interactions across different systems and this is most striking across immunology, hematology, and neuroscience. This reductionist approach does not allow understanding of the *in vivo* orchestrated response generated through integration of all systems. However, many factors make the understanding of multisystem cross-talk in response to a threat difficult, for instance the nervous and immune systems share communication molecules and receptors for a wide range of physiological signals. But, it is clear that physical, hard-wired connections exist between the two systems, with the key link involving sensory, unmyelinated nerve fibers (c fibers) containing the neuropeptide calcitonin gene-related peptide (CGRP), and modified macrophages, mast cells and other immune and host defense cells in various locations throughout the body. In this review we will therefore focus on the induction of CGRP and its key role in the neuroimmune axis.

## Sensory neurotransmitter CGRP

Sensory neurotransmitters have been extensively studied and their ability to affect different body functions has been shown in a range of studies. One of the main sensory neurotransmitters involved in immune function is CGRP. CGRP exemplifies a neuroimmune connector, since it is released at the site of stimulation, affecting immediate responses as well as mediating information flow to the rest of the nervous system. CGRP is a critical, highly expressed sensory signal, making it an important member of neuro-immune communication pathways. C fibers, the smallest diameter unmyelinated sensory neurons, are the main source of this neuropeptide. Their small diameter generates one of the lowest threshold response elements in the nervous system indicating their vital role. To date, this low threshold has placed them in the category of nociceptive neurons as they are the first to register damage/toxins through the pain pathway. This categorization is reinforced by the fact that c fibers express on their surface the transient receptor potential vanilloid 1 (TRPV1) which is a key responder to tissue damage. However, below the pain threshold, C fibers are likely to be playing a critical role in physiological systems and in particular, in host monitoring and activation of host defense and immune responses due to their low activation potential.

## CGRP release *in vivo*

**Figure 1:**
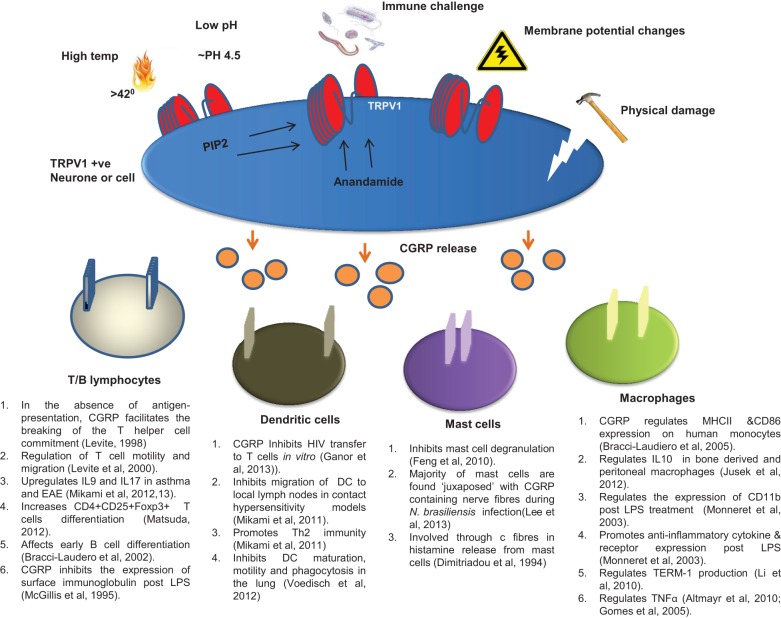
**Pathways of CGRP release via TRPV1 activation**. TRPV1 is activated by many external factors associated with pain but also regular sensation below pain thresholds. These include the well characterized responses to heat, pH, and damage, as well as to toxins, immune challenges and membrane potential changes brought about by physiological and pathological processes. Endogenous and/or intracellular activation can also occur through the actions of PIP2 and Anandomide. Any activation of TRPV1 results in release of CGRP from the neuron. This released CGRP then acts on its specific receptor on cells immediately adjacent to the neuron, which in the case of sensory c fibers can be lymphocytes, DCs, mast cells, macrophages or cells within the hemopoietic microenvironment of the bone marrow (not shown). The range of activities stimulated by CGRP are listed below each cell type.

CGRP is released in response to activation of TRPV1 in both the nervous and immune systems. In the nervous system, TRPV1 is expressed along the entire length of the sensory c fiber neurons, from the periphery to the somata in the CNS (Szallasi, 1995). These neurons innervate every organ and tissue in the body (Buck and Burks, 1986; Holzer, 1991; Szallasi and Blumberg, 1999). Although a key exogenous ligand for TRPV1 is capsaicin, TRPV1 is also activated by a range of other endogenous agonists including heat (>43°C) (Caterina et al., 1997; Tominaga et al., 1998), protons (~pH 4.5) (Vyklicky et al., 1998; Jordt et al., 2000), lipids like anandamide (Olah et al., 2001), phosphatidylinositol(4,5)-biphosphate (PIP_2_) (Chuang et al., 2001), and voltage (Gunthorpe et al., 2000) (summarized in Figure 1). Heat and low pH activate TRPV1 by distinct molecular recognition sites (Jordt et al., 2000). Additionally, TRPV1 has been suggested to be a mechanosensitive receptor in mediating nociceptive signals; for example TRPV1 deficient mice exhibit reduced sensitivity to post colorectal distension compared to controls (Jones et al., 2005) adding an interesting physiological element to TRPV1 activation. In rats, sensory vanilloid receptor-bearing nerves are classed as peptidergic and/or purinergic (Guo et al., 1999). Peptidergic nerves express TRPV1 and co-express neuropeptides whilst purinergic nerves express TRPV1 and co-express an adenosine triphosphate (ATP) gated ion-channel known as P2X_3_ (Yiangou et al., 2001).

Besides the well documented role of TRPV1 in the nervous system, we now know that it is also expressed on immune cells, which has direct relevance to this review. Absence of TRPV1 signaling has been shown variously to exacerbate inflammation (Massa et al., 2006; Huang et al., 2009) and these effects can be attributed indirectly to the subsequent lack of CGRP. Similarly, agonism of TRPV1 with capsaicin has been shown to inhibit differentiation and cytokine secretion of human dendritic cells (Toth et al., 2009), thereby reducing early activation signals on challenge.

There are two isoforms of CGRP known: αCGRP and β CGRP. αCGRP has been shown to be a potent vasodilator and exhibits marked synergism with inflammatory mediators to induce local edema (Bernard and Shih, 1990). αCGRP's receptors are found on mouse bone marrow cells, providing strong evidence toward an eminent effect for αCGRP in modulating immune cell function and differentiation which in turn may affect the inflammatory cascade in response to a pathological threat (Mullins et al., 1993). αCGRP has also been identified as the predominant isotype in sensory neurons in the dorsal root ganglion (DRG) (Gibson et al., 1988). Unless stated otherwise, the observations discussed below refer to αCGRP only.

## Physiological role for CGRP

CGRP was first described as a vasodilator nearly 30 years ago (Brain et al., 1985). It is now well-established that CGRP regulates blood pressure (Brain and Grant, 2004), is associated with the onset of migraines when at high levels (Olesen, 2011a,b), promotes the maintenance of mucosal integrity in the gastro-intestinal tract (GIT) (Szolcsanyi and Bartho, 2001), aids stem cell growth and maintenance *in vitro* (Dong et al., 2010) and modulates keratinocyte growth (Roggenkamp et al., 2013). CGRP plays a role in facilitating the absorption of intraluminal amino acids across the distal parts of the intestines (Mourad et al., 2009). In the lungs and elsewhere, CGRP promotes wound healing (Zhou et al., 2013). In the stomach, gastric acid stimulates the release of CGRP via activation of local afferent neurons. CGRP itself orchestrates the secretion of chemical factors that inhibit gastric acid secretion and induce muscle repair (Aihara et al., 2005, 2006). Furthermore, CGRP and other neuropeptide containing nerve fibers extend all the way to the epithelial cell lining of the gut, suggesting a role in monitoring of the external (gut) environment. An association with immune cells present in gut epithelium also suggests an important role in the immune responses generated there (Engel et al., 2010, 2011).

## CGRP and immunity

CGRP is the main neurotransmitter of the nociceptive sensory c fibers, but is also present in other sensory nerve fibers i.e., type A and B medium-sized neurons. CGRP is released when these nerves are activated by specific agonists of TRPV1 usually during trauma or injury and therefore additionally associated with pain perception. However, c fibers have also been implicated in non-nociceptive activities including modulation of immune responses, through neuropeptide release (Beresford et al., 2004; Shepherd et al., 2005a,b). In fact CGRP is abundant in many immune organs. Nerve fibers containing CGRP have been identified in bone marrow, thymus, spleen, lymph nodes, skin, lungs, and gut and CGRP receptors are found on many hematopoietic cell types (Santambrogio et al., 1993; Petitto et al., 1994; Mach et al., 2002). In the bone marrow, these fibers accompany noradrenergic sympathetic fibers and are distributed throughout the marrow. The role of sensory neurotransmitters in the hematopoietic process has been examined for CGRP. Treatment with capsaicin, a potent neurotoxin to c fibers and activator of TRPV1, produced a dramatic change in bone marrow hematopoiesis when measured by *in vitro* colony-forming assays suggesting that CGRP has direct access to hematopoietic progenitors (Broome et al., 2000). CGRP positive nerves have also been detected in the cortical and vascular regions of lymph nodes. No evidence has been obtained/presented for the presence of parasympathetic nerves in lymph nodes or for the presence of the parasympathetic neurotransmitter acetyl choline (Schafer et al., 1998). However, non-neural sources for acetyl choline in lymphatic organs have been identified (Rosas-Ballina et al., 2011) where it is secreted by resident memory T cells in the spleen facilitating vagal anti-inflammatory functions, therefore, playing part in the vagal-immune pathways.

Many studies have demonstrated specific roles for CGRP and other peptides, including substance P, in the generation of both pro and anti-inflammatory immune responses. CGRP in particular has been the focus of many studies trying to unveil its involvement in a range of inflammatory models and immune conditions e.g., diabetes (Morrison et al., 2009), sepsis (De Winter et al., 2009), EAE (Mikami et al., 2012a) Crohn's Disease (Smith and Smid, 2005), and ulcerative colitis (Engel et al., 2012; Li et al., 2013a). In rats, dextran sulfate sodium (DSS)-induced colitis worsened when treated with a CGRP antagonist suggesting a protective role for CGRP in colitis (Engel et al., 2010).

Once released, CGRP plays multifunctional roles at different sites by binding to its receptor calcitonin receptor like receptor (CRLR) and its receptor activity-modifying protein 1 (RAMP1) found on T and B lymphocytes (Mikami et al., 2012a,b), macrophages (Fernandez et al., 2001), mast cells (Eftekhari et al., 2013), and dendritic cells (Mikami et al., 2011) among others (See Figure 1). CGRP is released from afferent fibers at the site of stimulation, presenting a motor-like mode of action conducted in parallel with afferent signaling to the DRG (Holzer and Maggi, 1998). However, it is well documented that cells of the immune system, i.e., lymphocytes (Wang et al., 1999), monocytes (Bracci-Laudiero et al., 2005), and macrophages (Linscheid et al., 2004; Lee and Zhang, 2012) also synthesize CGRP. It is thought that CGRP operates through the intracellular molecules Protein kinase C (PKC), PKA, and MAP kinase (MAPK) (Li et al., 2013b; Zhou et al., 2013) particularly in the lung and uterus (Bai et al., 2012; Mikami et al., 2013; Wong et al., 2013). In addition, CGRP influences NF-kappa-B activation and induction of cAMP suppressor genes in immune cells, particularly dendritic cells (Harzenetter et al., 2007) thereby regulating function.

## CGRP regulates dendritic cell functions

Physiologically, CGRP was first found to be closely associated with dendritic cells (DC) in the skin (Hosoi et al., 1993). This study was the first to show a physical association between CGRP and Langerhans cells (LC) in the human epidermis. Functionally, it was one of the earliest publications to suggest an inhibitory function for CGRP on LC antigen presentation. So far, the majority of publications on the role of CGRP on DC suggest a predominant anti-inflammatory rather than pro-inflammatory effect. CGRP can modulate antigen presentation in DC (Mikami et al., 2011), and inhibit lipopolysaccharide induction of co-stimulatory signaling via the CD80 and CD28 receptors on dendritic cells and monocytes thereby affecting the function of T cells (Fox et al., 1997). Recently, Ganor et al. published a study on the role of CGRP during HIV-1 infection. Initially, CGRP plasma levels increased during the viral infection interfering with the induction of infection through LCs. CGRP inhibited the HIV-1 transfer to T cells, dampening the viral replication by 6 fold (Ganor et al., 2013). CGRP has also been shown to inhibit the migration of LC to local lymph nodes in contact hypersensitivity models, promoting Th2 type immunity in the process (Mikami et al., 2011). *In vitro* lung (DC) maturation was inhibited with CGRP (Rochlitzer et al., 2011). This correlated with a down regulation of the CGRP receptor on these DC during airway inflammation and these effects were reversed with the CGRP receptor antagonist hCGRP_8−37_. CGRP continued to influence the antigen presenting functions of DCs in the lungs even when DCs were pre-treated with CGRP. DC phagocytosis and motility were modulated by CGRP (Voedisch et al., 2012) suggesting an important role for CGRP in maintaining immune integrity in the lungs via the control of DC functions. Similar to its effects on DCs in the skin and lungs, CGRP also inhibits the function of bone marrow-derived DCs. Through its receptor complex, CGRP inhibited the production of (Tumor necrosis factor) TNFα and IL12 post-LPS treatment highlighting its potent anti-inflammatory roles (Tsujikawa et al., 2007). CGRP has additionally been identified as a potent inhibitor of TLR induced inflammatory agents TNFα and CCL4 (Harzenetter et al., 2007). CGRP has also been shown to down regulate the surface expression of HLA-DR and CD86 on mature and immature human DC (Carucci et al., 2000), suggesting a mechanism whereby CGRP may dampen DC-T cell interaction.

## CGRP regulates lymphocyte differentiation and cytokine production

CGRP shares aclose relationship with T cells. The interaction of T cell with DCs and macrophages, both of which express the CGRP receptor, is influenced by CGRP expression. (Ben-Horin and Chowers, 2008; Rochlitzer et al., 2011; Jusek et al., 2012; Holzmann, 2013). The capability of CGRP to regulate T cell function was proposed as early as 1988 (Umeda et al., 1988). In the last decade, studies have demonstrated a Th2 polarized T cell response after CGRP administration. Publications strongly suggest a TH2 preference by CGRP, through its influence on T cell differentiation (Levite, 2000; Ding et al., 2008). In more than one study, CGRP exposure to different DCs enhanced a TH2 type immunity increasing IL4 production while decreasing the TH1-associated cytokines interferon gamma (IFNγ) and IL2 (Wang et al., 1992; Tokoyoda et al., 2004; Mikami et al., 2011). The observed sensitization, according to Tokoyoda et al., occurs through the CGRP receptor activating cAMP/PKA pathways with the involvement of the conventional CD3/CD28 co-stimulation signal in T cells (Tokoyoda et al., 2004). CGRP has been shown to promote TH2 cytokine production *in vitro* and decrease TH1 cytokines (Levite, 1998, 2008). In 1998 and again in 2001, Levite et al., compared antigen-driven and neuropeptide-driven cytokine secretion from T helper cells (Levite, 1998; Levite and Chowers, 2001). They found that in a controlled *in vitro* environment TH0, TH1, and TH2 cells all produced “unconventional” cytokines respective to their phenotype when exposed to neuropeptide, with TH1 secreting IL4, TH2 secreting IFNγ, and TH0 secreting both. This suggests an ability to break the T helper line commitment and regulation of T cell functions. CGRP also up regulated IL9 and IL17 in both asthma and experimental autoimmune encephalomyelitis (EAE) models (Mikami et al., 2012a, 2013). IL17 has been attributed to a range of inflammatory functions and roles; however, its association with CGRP is still not understood. In an EAE model, IL17 levels dropped in RAMP-1^−/−^ mice and TH17 functions were suppressed, suggesting a role for CGRP in the TH17 induced EAE (Mikami et al., 2012a). This has since been corroborated in models of psoriasis where TH17 cells are the dominant inflammatory phenotype (Ostrowski et al., 2011). In contrast, the association of CGRP with CD4+CD25+Foxp3+ cells has also been studied in a model of EAE (Matsuda et al., 2012). CGRP transfected DCs were able to increase differentiation of CD4+CD25+Foxp3+ regulatory cells (Matsuda et al., 2012). CGRP, is also produced by B lymphocytes in inflammatory conditions under the influence of nerve growth factor (NGF) (Bracci-Laudiero et al., 2002). The effect of CGRP on early B cell differentiation has been examined using a pre-B cell line population (70Z/3). CGRP inhibited the expression of surface immunoglobulin in response to LPS (McGillis et al., 1993, 1995). These studies suggest a crucial role for CGRP in early B cell differentiation, a finding supported by later studies (Fernandez et al., 2000; Schlomer et al., 2007).

## CGRP and immune motility, migration, and adhesion

In 1992, CGRP was described as a neuropeptide with “chemotactic” properties on human CD4 and CD8 T lymphocytes in the skin, inducing T cell trafficking (Foster et al., 1992). Ever since, T cells more than any other cell, have been the main targets for studies to understand the effect of CGRP on immune cell migration, adhesion, and motility. CGRP plays a key role in T cell adhesion to fibronectin (Levite, 1998; Levite et al., 1998) and beta integrin mediated T cell migration. Somatostatin, CGRP and neuropeptide Y all induced of freshly purified T cells to fibronectin coated plates (Springer et al., 2003). Deletion of CGRP results in a sustained decrease in leukocyte circulation and migration, highlighting its role in the bone marrow and in the mobilization of immune cells (Broome and Miyan, 2000; Broome et al., 2000). In the gut CGRP from c fibers stimulates T cell migration (Talme et al., 2008). *In vitro* studies showed that in contrast to other neuropeptides, CGRP can stimulate the migration of CD3 T cells into collagen matrix (Talme et al., 2008), an effect inhibited with CGRP receptor antagonist. In the absence of other immunological signals extracellular K(+) plays a critical role in stimulating T cell-integrin induced adhesion and migration. T cell voltage-gated K channels (Kv1.3) are targeted by a number of molecules to control T cell-integrin induced motility (Levite et al., 2000). CGRP binding to T cells, through its receptor, opens the voltage-gated K(+) channels, releasing K(+) from the intracellular matrix and activating β 1 integrin. This facilitates T cell integrin-induced function highlighting a critical role for CGRP in alternative pathways for T cell adhesion, migration and motility. The effect of CGRP on macrophage and monocyte motility and chemotactic activity has also been studied. *In vitro*, inoculated promastigotes from cutaneous *Leishmania major*, demonstrated that CGRP, substance P, and somatstatin had regulatory effects on macrophage chemotactic activity (Ahmed et al., 1998; Han et al., 2010). CGRP was suggested to play an important role in human monocyte adhesion and migration (Linscheid et al., 2004). Human neutrophils cultured *in vitro* with CGRP and LPS secreted high levels of the chemokine IL8 (He et al., 2002), whilst CGRP has also been proposed to stimulate eosinophil migration (Dunzendorfer et al., 1998). CGRP increased the adhesion-related DC CD103+ ligand epithelial cadherin (E-cadherin) expression in human bronchial epithelial cells (Bai et al., 2012).

## Regulation of other immune cells

A wealth of literature can be found on the role of CGRP in the inhibition of mast cell degranulation. Many studies have reported mast cells and CGRP fibers “juxtaposed” in different models (Bienenstock et al., 1987; Lee et al., 2013). Controlled low dose capsaicin has been used to induce the release of CGRP from sensory nerves (Demirbilek et al., 2004). It has been estimated that nearly 60–70% of mast cells in the jejunum of rats infected with *Nippostrongylus brasiliensis* are juxtaposed to CGRP containing nerve fibers suggesting a role for this association in the immune response to this infection at least (Bienenstock et al., 1987; Dimitriadou et al., 1994) but no doubt in others also. Moreover, desensitization or deletion of c fibers with high doses of capsaicin severely impaired mast cell recruitment during *Schistosoma mansoni* infection in mice (De Jonge et al., 2003; Van Nassauw et al., 2007). Similarly, CGRP inhibited mast cell degranulation in a model of cerebral ischemia in rats (Feng et al., 2010) and in response to anti/IgE treatments, in comparison to other neuropeptides, in an *in vitro* assay with primary human mast cells (Kulka et al., 2008).

CGRP receptor is also expressed on human monocytes (Caviedes-Bucheli et al., 2008). Under the influence of NGF, CGRP has been shown to regulate the expression of MHCII, CD86 and the production of IL10 in human monocytes *in vitro* (Bracci-Laudiero et al., 2005). However, it seems that the role of CGRP in monocytes/macrophages seems to be less direct than that seen in other immune cells. CGRP is synthesized by monocytes/macrophages and can regulate immune functions of these cells (Ma et al., 2010). For instance CGRP can regulate CD11b expression after LPS exposure in human macrophages and neutrophils promoting anti-inflammatory functions (Monneret et al., 2003). Recently, macrophages have been studied in the context of pain induction and their involvement in pain pathways during inflammation (Hasegawa-Moriyama et al., 2013; Isami et al., 2013; Taves et al., 2013). In various studies, CGRP treatment increased IL10 from both peritoneal and bone marrow derived macrophages in response to LPS (Jusek et al., 2012). CGRP receptor absence resulted in the influx of neutrophils and inflammatory mediators into the peritoneal cavity (Jusek et al., 2012). CGRP was able to regulate the synthesis of triggering receptor expressed on myeloid cells-1 (TERM-1) in LPS-induced macrophages (Li et al., 2010). This effect was absent without LPS. However, the main contribution of CGRP to macrophage function is likely to incorporate its newly found links to the pro-inflammatory cytokine TNFα. This is the main cytokine released by macrophages in response to pathological challenges.

## CGRP and TNFα: a new chapter

TNFα and CGRP are two important molecules that demonstrate a key bi-directional influence between the nervous and immune systems. TNFα functions through its receptors TNFRI and TNFRII. These receptors are found on glial cells in the spinal cord which, when activated, release prostaglandins, activating in turn nociceptive neurons and eliciting a pain response. Thus, the involvement of TNFα in peripheral and central pain is no longer a matter of doubt (Saito et al., 2010; Zhang et al., 2010a; Bressan et al., 2011; Gim et al., 2011; Teodorczyk-Injeyan et al., 2011). TNFα has been associated with the enhancement of trigeminal neuronal sensitivity to capsaicin in rats (Khan et al., 2008); exposure to TNFα for 5 min doubles the sensitivity of fura-loaded trigeminal neurons to capsaicin via TRPV1. Both TNFα receptors were present on capsaicin-sensitive trigeminal neurons co-expressed with TRPV1 (Khan et al., 2008). The presence of the TNFα receptors next to TRPV1 suggests that TNFα sensitizes TRPV1 positive neurons through its receptors. TNFRI plays a role in sensitizing tetrodotoxin (TTX)-resistant sodium channels (Jin and Gereau, 2006). It is likely that TNFRI has additional functions in immediate hyperalgesia and nociception to damage (Sommer et al., 1998), whileTNFRII is thought to have a role in nociception in persistent/chronic injurious conditions (Constantin et al., 2008; Schafers et al., 2008). A recent study has also linked TNFα to meningeal nociception (Zhang et al., 2010b), where it was suggested that TNFα functioned through TNFRI & TNFRII on nociceptive neurons and was associated with the “throbbing” sensation significant in migraines. Interestingly, neutralizing antibodies against TNFRI modulated thermal and mechanical hyperalgesia while antibodies against TNFRII had no affect (Sommer et al., 1998). Electrophysiological techniques showed that the local application of TNFα at the peripheral level of sensory neurons excites TRPV1 bearing sensory c fibers suggesting a specific role for TNFα in nociception (Sorkin and Doom, 2000). Additionally, TNFα increased CGRP release from sensitized capsaicin sensitive neurons when given 5 min prior to capsaicin treatment in comparison to vehicle treated rats (Khan et al., 2008). During LPS endotoxin treatment the increase in CGRP release was attributed to TNFα sensitizing afferent neurons in the presence of the prostaglandin sub types, prostanoids (Hua et al., 1996). These studies suggest that the nociceptive functions of TNFα occur via the specific capsaicin receptor TRPV1.

The activation of the specific capsaicin receptor TRPV1 located on c fibers or capsaicin sensitive neurons results in the release of sensory neuropeptides i.e., CGRP and SP (Helliwell et al., 1998; Bhave et al., 2002; Shepherd et al., 2005a; Ren et al., 2011). Additionally, reduction in CGRP tissue expression levels was seen in the dorsal root ganglia of rats injected with Entanercept (anti-TNFα treatment) (Horii et al., 2010). In both rats and humans with rheumatoid arthritis, CGRP and SP levels were reduced by 50% in the serum after treating with Etanercept (Origuchi et al., 2010). By contrast, TRPV1 activation via capsaicin and SA13353 (capsaicin analog) attenuated LPS-induced TNFα release in serum, and this effect was partially inhibited with CGRP antagonist treatment and in TRPV1^−/−^ or sensory denervated mice (Tsuji et al., 2009) suggesting a regulatory function for CGRP on TNFα i.e., CGRP is able to downregulate TNFα *in vivo*. Both small doses (1 mg/kg) and large doses (150 mg/kg) of the TRPV1 agonist capsaicin have been found to downregulate serum levels of a range of pro-inflammatory cytokines including TNFα (Demirbilek et al., 2004), and the effects of capsaicin are attenuated in the presence of the antagonist for either TRPV1 or CGRP (Peng and Li, 2009).

In Toll-like receptor stimulated dendritic cells, CGRP causes a rapid up-regulation of inducible cAMP early repressor (ICER) which competes with ATF-2 for binding at the *Tnfα* promoter gene preventing gene expression of TNFα (Altmayr et al., 2010). In experiments using a non-irritating capsaicin analog (Vanillyl nonanoate) to examine the protective role capsaicin has on gastric mucosa (Luo et al., 2011), increased release of CGRP via TRPV1 activation had the ability to attenuate TNFα release in the serum post ethanol treatment. Blocking CGRP release from TRPV1 caused an increase in TNFα serum levels and increased gastric mucosal damage (Luo et al., 2011). Taken together all the findings suggest the presence of a TNFα-CGRP regulatory “loop” that is mediated via TRPV1 bearing sensory neurons releasing CGRP and/or through intracellular transcriptional interference of TNFα expression by immune cells.

## Conclusion

CGRP has critical and wide-ranging control functions in various body systems and is particularly critical to normal hemopoietic and immune functions. CGRP-containing c nerve fibers are found everywhere in the body and are associated, in many locations, with specific immune cells including dendritic cells, mast cells and T cells. It appears to be the key mediator of neuro-immune communication with the c fibers acting as both sensory pathways, informing the nervous system of peripheral challenges, and as a local controller of immune functions. Knocking out this neural system results in major down-regulation of bone marrow hemopoietic output supporting a role for these nerve fibers in sensory feedback to immune control centers, already identified in the central nervous system by Denes et al. (2005). It is therefore becoming increasingly clear that this intimate connection between a specific subset of specialist sensory neurons and immune cells throughout the body, together with the connections to the brain and host defense control centers, is vital to the integrated, coordinated host defense response to any peripheral challenge. Moreover, it is clear that immune mediators, specifically pro-inflammatory cytokines IL-1a and TNFα, have direct effects on brain activity and whole body responses through the stimulation of specific circuits involved in so-called sickness behaviors (Dantzer and Kelley, 2007) that channel energy into host defense at the cost of social and other activities. A more complete understanding of this neural-immune interaction is vital to uncover key avenues for intervention to override abnormal functions and improve normal functions.

### Conflict of interest statement

The authors declare that the research was conducted in the absence of any commercial or financial relationships that could be construed as a potential conflict of interest.
